# ^17^O NMR Spectroscopy: A Novel Probe for Characterizing Protein Structure and Folding

**DOI:** 10.3390/biology10060453

**Published:** 2021-05-21

**Authors:** Srinivasan Muniyappan, Yuxi Lin, Young-Ho Lee, Jin Hae Kim

**Affiliations:** 1 Department of New Biology, Daegu Gyeongbuk Institute of Science and Technology (DGIST), Daegu 42988, Korea; srinivasan@dgist.ac.kr; 2 Research Center for Bioconvergence Analysis, Korea Basic Science Institute, Cheongju 28119, Korea; linyuxi@kbsi.re.kr; 3 Department of Bio-Analytical Science, University of Science and Technology, Daejeon 34113, Korea; 4 Graduate School of Analytical Science and Technology, Chungnam National University, Daejeon 34134, Korea; 5 Research Headquarters, Korea Brain Research Institute, Daegu 41068, Korea

**Keywords:** ^17^O NMR spectroscopy, protein structures, protein folding, oxygen-17

## Abstract

**Simple Summary:**

Oxygen is one of the most abundant atoms in the body. Biomolecules, including most proteins, contain a significant number of oxygen atoms, contributing to the maintenance of the structural and functional integrity of biomolecules. Despite these favorable attributes, detailed characterization of these atoms has been challenging, particularly because of the lack of an appropriate analytical tool. Here, we review recent developments in biomolecular ^17^O nuclear magnetic resonance spectroscopy, which can directly report the physicochemical properties of oxygen atoms in proteins or related biomolecules. We summarize recent studies that successfully employed this technique to elucidate various structural and functional features of proteins and protein complexes. Finally, we discuss a few promising benefits of this methodology, which we believe ensure its further development as a novel and powerful tool for investigating protein structure and folding.

**Abstract:**

Oxygen is a key atom that maintains biomolecular structures, regulates various physiological processes, and mediates various biomolecular interactions. Oxygen-17 (^17^O), therefore, has been proposed as a useful probe that can provide detailed information about various physicochemical features of proteins. This is attributed to the facts that (1) ^17^O is an active isotope for nuclear magnetic resonance (NMR) spectroscopic approaches; (2) NMR spectroscopy is one of the most suitable tools for characterizing the structural and dynamical features of biomolecules under native-like conditions; and (3) oxygen atoms are frequently involved in essential hydrogen bonds for the structural and functional integrity of proteins or related biomolecules. Although ^17^O NMR spectroscopic investigations of biomolecules have been considerably hampered due to low natural abundance and the quadruple characteristics of the ^17^O nucleus, recent theoretical and technical developments have revolutionized this methodology to be optimally poised as a unique and widely applicable tool for determining protein structure and dynamics. In this review, we recapitulate recent developments in ^17^O NMR spectroscopy to characterize protein structure and folding. In addition, we discuss the highly promising advantages of this methodology over other techniques and explain why further technical and experimental advancements are highly desired.

## 1. Introduction

Our bodies are mainly composed of several biomolecules, including proteins, nucleic acids, polysaccharides, and lipids, along with a large amount of water [1]. These biomolecules maintain complex yet delicately balanced interactions to modulate nearly infinite physiological processes within the body. Among these biomolecules, proteins are essential biological macromolecules that perform a wide range of functions such as structural support for the cells, defense against foreign molecules, cellular communication, and catalytic activity facilitating chemical reactions [2]. These functionalities of proteins are directly related to their three-dimensional structural features. The atomic-resolution structure determination of proteins is one of the most intriguing and important issues to be addressed by modern biology [3]. To date, numerous structural features of proteins have been elucidated using diverse multidisciplinary techniques. Among these techniques, X-ray crystallography, nuclear magnetic resonance (NMR) spectroscopy, cryo-electron microscopy, or their combinatory applications have contributed significantly to our understanding of the structural features of various proteins and protein–biomolecule complexes [4,5,6,7,8]. These techniques complement each other; however, NMR spectroscopy has a unique position here owing to its evident advantages and limitations [7]. 

Traditionally, the NMR techniques for observing ^1^H, ^13^C, and ^15^N nuclei have been utilized to obtain structural information about proteins with selective isotopic labeling of respective nuclei. The sole or combined NMR methodologies for the ^1^H, ^13^C, and ^15^N nuclei have made a tremendous impact on studying the structure and dynamics of proteins [9,10,11]. Although hydrogen, carbon, and nitrogen are undoubtedly the major constituents of the body, the most frequently observed atoms are oxygen [12]. It is estimated that 65% of the total human body mass comes from oxygen (carbon, hydrogen, oxygen, and nitrogen atoms constitute more than 95% of the human body mass), indicating that understanding the chemical and biological features of oxygen is critical for appreciating the structural and biological features of biomolecules [12]. It is also noteworthy that one of the most important non-covalent forces for maintaining the structural integrity of biomolecules and mediating biomolecular interactions is the hydrogen bond, a significant portion of which involves oxygen atoms. For example, structural units of nucleic acids, such as base pairing in DNA and RNA helical structures and tetraloop structures of RNAs, involve many oxygen-mediating hydrogen bonds [13,14]. Most secondary structures found in proteins, such as α-helices and β-sheets, are formed from at least one oxygen-mediated hydrogen bond per residue. Tertiary and quaternary structures of proteins are also frequently stabilized by extensive hydrogen bond networks, the perturbation of which often results in misfolding and loss of proteostasis mechanisms. The catalytic centers of many enzymes often comprise oxygen-containing functional groups, which mediate protein –substrate interactions or facilitate enzymatic reactions. 

Therefore, NMR studies on oxygen nuclei have a great potential to provide additional information about biomolecular structures and interactions. However, the NMR-active stable isotope for oxygen is ^17^O, and it has a very low natural abundance of only 0.037% (more predominant oxygen nuclei, ^16^O and ^18^O, are NMR-silent) [15,16,17]. In addition, ^17^O has quadrupolar nuclei with a spin quantum number of I = 5/2, and quadrupolar nuclei often show much larger quadrupolar interactions. This results in severe line broadening in the ^17^O NMR signals, especially when measured in the solution state, owing to its free molecular tumbling motions in the solution. 

Several notable trials have been conducted to circumvent these technical challenges of ^17^O NMR spectroscopy. First, it was shown that severe line broadening in ^17^O NMR signals could be circumvented to some extent by employing magic angle spinning (MAS) solid-state NMR technologies [16]. In addition, modern superconducting magnets and probes with novel developments in ^17^O-labeling protocols have enabled us to record high-quality ^17^O NMR spectra even from relatively large protein complexes in either a liquid or a solid state [17]. Notably, over the decades following a few initial trials, the use of solution-state ^17^O NMR methodology for investigating large biomolecules was not preferred, based on the misconception that ^17^O signals might not exhibit sufficient sensitivity and resolution for large macromolecules in a liquid state due to efficient ^17^O quadrupole relaxation. However, recent reconsideration of the theoretical framework of nuclear quadrupole relaxation, along with further advanced instrumental development of NMR methodology, has enabled us to record the solution-state ^17^O NMR signals of large biomolecules with sufficient sensitivity and resolution [18,19]. When a half-integer quadrupole nucleus is placed under an ultra-high magnetic field, it shows three distinct exponential components: the central transition (CT), the first satellite transition (ST_1_), and the second satellite transition (ST_2_). The relaxation theory predicts that the transverse relaxation rate for ST_1_ and ST_2_ increases monotonically with ω_o_τ_c_. However, although the transverse relaxation rate for CT first increases with ω_o_τ_c_, it reaches a maximum at ω_o_τ_c_ = 1, and subsequently decreases again in the regime of ω_o_τ_c_ > > 1 [19]. This indicates that the corresponding CT signal can be narrow under a slow-motion condition, and that it may be feasible to obtain high-resolution signals for half-integer quadrupolar nuclei, such as ^17^O, of a large slow-tumbling biomolecule, even in an aqueous solution [18,19].

Herein, we briefly summarize the developments and applications of biomolecular ^17^O NMR spectroscopy with regards to characterization of protein structure and misfolding and discuss its future directions. In particular, we focus on recent contributions of ^17^O NMR spectroscopy to reveal structural features, related functions, and folding processes of various proteins. As Gang Wu and his colleagues published a comprehensive review of recent developments in ^17^O NMR spectroscopy on organic and inorganic molecules [17], we concentrate on discussing recent trials to characterize the structure and misfolding of proteins and protein complexes, as well as their physiological implications. 

## 2. Solution- and Solid-State ^17^O NMR as a Probe for Studying Protein Structure

**Figure 1:**
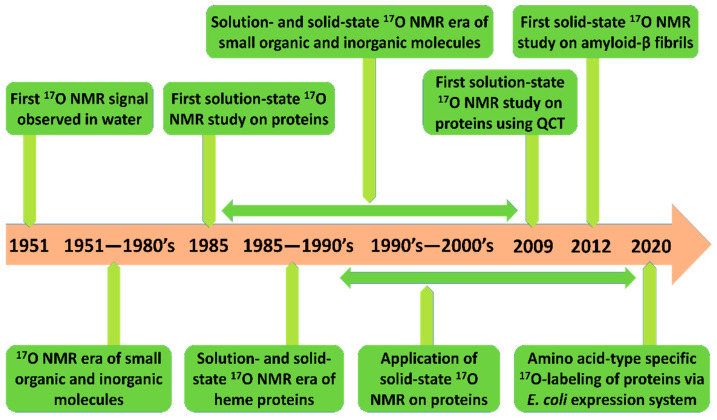
The timeline of notable developments in the field of ^17^O nuclear magnetic resonance (NMR) spectroscopy for biomolecular investigations [15,17,20].

Novel advancements in ^17^O NMR techniques provide unprecedented means for probing site-specific inter- and intra-molecular interactions of macromolecules in both aqueous and solid-state conditions. In this section, we briefly recapitulate notable developments and applications of ^17^O NMR spectroscopy for studying biological macromolecules. Figure 1 summarizes the developments and timeline of ^17^O NMR studies; particular marks have been added to studies regarding protein structure and folding. 

### 2.1. Early Solution-State ^17^O NMR-Based Studies for Biomolecules

In 1951, Alder and Yu [21] observed the first ^17^O NMR signal with several oxygen-containing solvents such as water, methanol, ethanol, and acetic acid. Since then, the ^17^O NMR technique has established its position over two decades as a superior and unique tool for examining the physical and chemical properties of various organic and inorganic compounds [15,17,22]. In 1983, Wisner et al. [23] first applied solution-state ^17^O NMR to study adenylate kinase enzyme–substrate complexes. However, their solution-state ^17^O NMR spectra were severely broad because of the highly efficient quadrupolar relaxation of the ^17^O nuclei. In the same year, Lee et al. [24] collected the solution-state ^17^O NMR spectra of the C^17^O-bound forms of ferrous horseradish peroxidase isozymes A and C and ferrous chloroperoxidase at a pH range of 4.5–9.5. From these analyses, all three proteins were identified to exist in two distinctive forms, acidic and basic, which also experienced reversible acid-base-induced transitions. This suggests that the same ionizable groups might have been involved in the acid-base transition processes of all three proteins. In addition, their results clearly demonstrated that the acidic form exhibited a ^17^O NMR signal at approximately 7 ppm up-field compared to that of the basic form. Moreover, they measured the exchange rate between the acidic and basic forms of the peroxidases and found that the exchange took place on a millisecond time scale. Interestingly, they also acknowledged that the exchange rate was faster for the CO-bound chloroperoxidase than for the CO-bound horseradish peroxidase isozymes A and C, implying that the CO-bound chloroperoxidase was more flexible and had a different proximal configuration around the heme cofactor. In addition, Lee and coworkers [25] measured the solution-state ^17^O NMR spectra of the C^17^O-bound forms of sperm whale myoglobin, human hemoglobin (hHbC^17^O), and rabbit hemoglobin (rHbC^17^O) at 8.45 and 11.7 T, from which they were able to obtain relatively narrow ^17^O NMR signals. Two well-resolved ^l7^O NMR signals were identified in these observations, each of which originated from the α and β chains of rHbCO. However, hHbCO signals from the α and β chains were indistinguishable. This implied that the chemical environments of the C^17^O ligands bound to the α and β chains of hHbCO were similar, while the C^17^O ligands bound to the α and β chains of rHbCO were different.

Subsequently, the binding of the dioxygen (O_2_) molecule, one of the most important ligands for heme-containing proteins (hemoproteins), was investigated using solution-state ^17^O NMR spectroscopy, in which more physiological behaviors of proteins could be visualized. In 1989, Gerothanassis et al. [26] attempted to obtain ^17^O NMR signals from several synthetic oxygenated hemoprotein models in aqueous conditions. Although they could observe two well-resolved signals from the heme-bound ^17^O_2_ molecules, rigorous measurements of chemical shifts and electric field gradient tensors were not amenable. Furthermore, the exchange rate of O_2_ was higher in solution, hindering spectral measurements over a wider range of temperatures than that in solid-state NMR approaches. On the other hand, Oldfield and coworkers [27] obtained the first solid-state ^l7^O NMR spectra of oxygenated heme group model, oxy-myoglobin, and oxyhemoglobin. Their results revealed that the ^17^O NMR spectra of all three model systems were highly similar at 77 K; additionally, they could obtain information about the oxygen rotation and estimate the Fe-O-O bond angle. 

### 2.2. Solid-state ^17^O NMR-Based Approaches

Owing to its several advantages over the solution-state NMR approaches, significant efforts have been devoted to the technical development of solid-state ^17^O NMR spectroscopy, which has enabled researchers to obtain high-quality solid-state ^l7^O NMR spectra from small organic molecules as well as large biological macromolecules [18,28,29,30,31,32,33,34,35,36,37,38,39,40]. A number of initial trials of solid-state ^17^O NMR spectroscopy have focused on investigating the structural features and dynamics of small molecules such as organic, inorganic, crystalline amino acids, short polypeptides, and nucleotide units [41,42,43]. After a decade-long accumulation of initial studies along with technical developments, in 2004 Lemaître and coworkers [44] measured solid-state ^17^O NMR spectra from a transmembrane peptide, which was synthetically labeled with ^17^O and then introduced into hydrated vesicles. They confirmed that this approach was effective enough to accurately follow subtle changes in C=O bond length. Furthermore, Hu et al. [45] synthesized ^17^O-[D-Leu10]-labeled gramicidin A and incorporated it into a biomimetic lipid bilayer environment. They could align their proteins by reconstructing lipid bilayers between 30 μm thick glass slides, from which static solid-state ^17^O NMR spectra were obtained to estimate the isotropic/anisotropic chemical shift and quadrupolar coupling information from the carbonyl ^17^O of D-Leu10. Subsequently, the static solid-state ^17^O NMR spectra of ^17^O-[D-Leu10]-gramicidin A were compared in the presence and absence of K^+^ ions, and a 40-ppm signal shift was observed, which was attributed to ~40% occupancy of K^+^ ions. This study demonstrated that ^17^O NMR spectroscopy is a highly sensitive tool for monitoring the physical and chemical states of membrane proteins. In 2008, Wong et al. [46] characterized the phospholemman (PLM) transmembrane domain using solid-state ^17^O MAS NMR spectroscopy at a low (less than 40%) ^17^O enrichment level and high lipid/peptide ratio (25:1). They analyzed the ^17^O MAS NMR signal line-shape of a ^17^O-glycine residue in the transmembrane region and found that the spectral features could not be explained by one symmetric oxygen atom in the glycine residue. As PLM constitutes a tetrameric complex in a lipid bilayer membrane condition, they concluded that the rotational symmetry of this complex may be *C*_2_ or *C*_1_ to the lipid bilayer axis. 

More recently, technical and methodological advancements in solid-state ^17^O NMR techniques have facilitated their application in challenging yet biologically important issues. In 2010, Zhu et al. [47] obtained high-quality solid-state ^17^O MAS NMR spectra for two robust protein–ligand complexes, the egg-white avidin–[^17^O_2_]biotin complex (the estimated size: 64 kDa) and the ovotransferrin–Al^3+^–[^17^O_4_]oxalate complex (OTf-Al^3+^-[^17^O_4_]oxalate; 80 kDa), at 21 T with 90% ^17^O enrichment. This work opened up a new possibility for using solid-state ^17^O NMR applications to study protein–ligand complexes as large as 300 kDa per ligand. Tang et al. [48] explored the utility of solid-state ^17^O MAS NMR for studying the highly unstable acyl-enzyme intermediates of chymotrypsin. This work was the first attempt to trap the highly unstable acyl-enzyme intermediates of a serine protease by quickly freeze-drying the solution and then performing solid-state ^17^O NMR measurements. They analyzed the ^17^O NMR spectra for three acyl-enzyme intermediates, all of which showed significant impacts on the ^17^O chemical shift due to the different hydrogen bonding environments in the oxyanion hole in the acyl-enzyme intermediates. 

So far, the application of ^17^O NMR to biological molecules that are solely enriched with ^17^O has been discussed. However, similar to solid-state ^13^C and ^15^N NMR heteronuclear correlation spectroscopy, heteronuclear couplings between ^17^O and ^15^N can be demonstrated by the use of ^17^O REDOR and ^15^N REAPDOR-type experiments. Gullion et al. [49] used a ^13^C-^17^O REAPDOR technique to measure intermolecular distances in the parallel and antiparallel β-sheet structures of tripeptides (L-alanyl-alanyl-alanine) that were site-specifically labeled with ^13^C and ^17^O. Hung et al. [50] also employed ^17^O-^15^N REDOR and ^15^N-^17^O REAPDOR techniques to experimentally determine the ^13^C-^17^O and ^15^N-^17^O dipolar couplings with glycine and uracil model molecules that were isotopically labeled with ^13^C, ^15^N, and ^17^O. More recently, Antzutkin et al. [51] applied ^15^N-^17^O REAPDOR to study a hydrogen bonding network in amyloid-β (Aβ) fibrils. They synthesized two selectively ^15^N- and ^17^O-labeled Aβ fragments, namely, Ac-Aβ_(16–22)_-NH_2_ (Ac-KLV_18_(^17^O)FF_20_(^15^N)AE-NH_2_) and Aβ_(11–25)_ (EVHHQKLV_18_(^17^O)FFA_21_(U–^13^C,^15^N)EDVG) amyloids. They observed that two different fibril structures could be formed depending on the pH of the incubated solution. At a low pH (pH 2.4), the antiparallel β-sheet structure adopted the 17+k ↔ 22–k register (k = −3, −2, …, 8), whereas at a high pH (pH 7.4) it exhibited a register of 17+k ↔ 20–k (k = −5, −4, …, 8). Further, they calculated the inter-strand C = ^17^O ··· H-^15^N hydrogen bond distances and showed a typical O ··· N distance of 2.7 Å. Subsequently, Wei et al. [52] investigated the polymorphisms exhibited by Aβ peptides, using the solid-state ^15^N REAPDOR and advanced mass spectrometry techniques. 

### 2.3. Recent Developments in Solution-State ^17^O NMR Spectroscopy for Large Proteins

**Figure 2:**
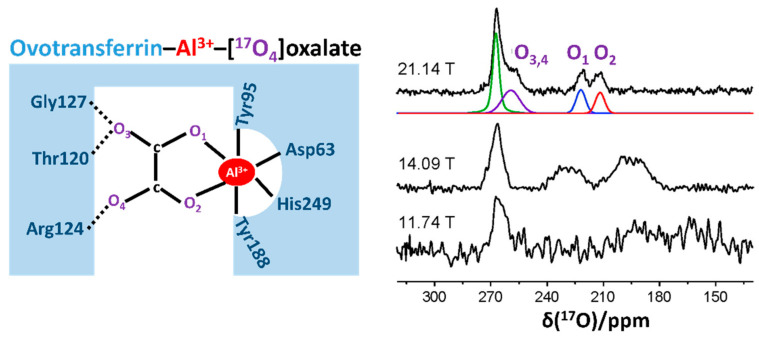
Schematic representation of the oxalate binding site in the ovotransferrin (OTf)–Al^3+^ complex (left panel), and the solution-state ^17^O quadruple central transition NMR spectra of [^17^O_4_]oxalate bound to OTf-Al^3+^ at different magnetic fields of 21.14 T, 14.09 T, and 11.74 T, respectively (right panel). The deconvoluted ^17^O signals for the spectrum at 21.14 T are also represented in distinctive colors. The figure was reproduced with permission from [18].

In 2009, Zhu et al. [18] applied a quadrupole central transition ^17^O (^17^O QCT) NMR technique and obtained high-resolution ^17^O NMR spectra from ^17^O-labeled palmitic acid bound to human serum albumin (66 kDa) and ^17^O-labeled oxalate bound to ovotransferrin (OTf-Al^3+^-[^17^O_4_]oxalate) in aqueous solution. Notably, the ^17^O QCT NMR signal was significantly narrower at 21.14 T than that at 11.74 T. From this study, they found that six molecules of palmitic acid could bind to HSA in a deprotonated state. They also acknowledged that the two oxygen atoms (O_1_ and O_2_ in Figure 2) maintained asymmetric interatomic distances with the aluminum center of OTf, as indicated by two separate ^17^O signals and their different parameters (Figure 2). This work well exemplified that ^17^O NMR is a versatile and powerful tool for characterizing various types of bonding interactions involving oxygens. Moreover, this study opens up new possibilities for the use of solution-state ^17^O QCT NMR applications to study reasonably large protein–ligand complexes at sub-millimolar concentrations under a high magnetic field. Notably, in their subsequent work on solid-state ^17^O MAS NMR study for the OTf-Al-[^17^O_4_]oxalate complex, they confirmed that the solid-state NMR data were highly consistent with the solution-state data, indicating that this protein–ligand complex sustained similar structural states regardless of their phase [47]. Subsequently, the authors extended a comprehensive ^17^O QCT NMR study to three ligand–protein complexes whose molecular sizes were even larger than those used in their previous trials, such as avidin-[^17^O_2_] biotin, OTf-Al-[^17^O_4_]oxalate, and pyruvate kinase-Mg-ATP-[^17^O_4_]oxalate (a tetrameric complex, total ~240 kDa) [19]. In addition, they determined the value of τ_c_ for the ligand–protein complexes at 298 K, and the observed value τ_c_ was in qualitative agreement with the theoretical prediction. Furthermore, they suggested that ^17^O QCT NMR can be applied to even larger proteins or protein complexes up to 400–500 kDa [19].

**Figure 3:**
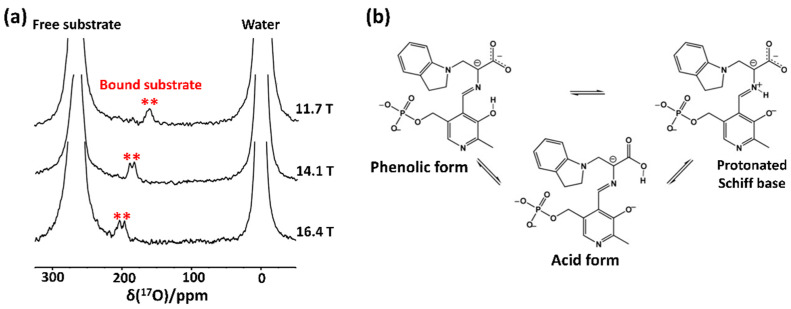
^17^O QCT NMR approach for the E(Q_3_)_indoline_ intermediates of tryptophan synthase. (**a**) The solution-state ^17^O QCT NMR spectra of the E(Q_3_)_indoline_ intermediate in tryptophan synthase measured at different magnetic fields of 16.4 T, 14.1 T, and 11.7 T, respectively. The ^17^O signals from the enzyme-bound intermediates are marked with red asterisks. (**b**) Illustration of exchange between tautomeric forms of the E(Q_3_)_indoline_ quinonoid intermediate. The figure was reproduced with permission from [54].

In 2013, Hanashima et al. [53] applied the solution-state ^17^O-NMR approach to detect the oxidized side chain of cysteine residue from a human Cu, Zn-superoxide dismutase. They specifically oxidized a cysteine thiol side chain by applying ^17^O_2_-gas to the protein sample and found from the resultant ^17^O NMR spectra that cysteine oxidation caused structural and dynamical changes in this protein. This method of ^17^O enrichment can be applied to various proteins whose Cys or Met residues are prone to oxidative stress. Recently, Young et al. [36] applied solution-state ^17^O QCT NMR to study the enzymatic intermediates of tryptophan synthase (143 kDa), a pyridoxal 5′-phosphate-dependent enzyme that mediates the biosynthesis mechanism of L-tryptophan. They measured ^17^O QCT spectra of the E(Q_3_)_indoline_ intermediate, which were formed by supplying [^17^O]-L-Ser as a substrate, at multiple magnetic strengths of 11.7, 14.1, and 16.4 T (Figure 3). They observed a field-dependent shift of ^17^O signals from the bound substrate and also identified two signals for the distinctive oxygen atoms from the carboxylate group of this intermediate (Figure 3a). Intriguingly, they acknowledged that the extracted isotropic chemical shifts of these signals were out of the expected range; they were up-field shifted, indicating more electronic shielding effects around the carboxylate group of E(Q_3_)_indoline_. Prior to this work, it had been proposed that the acidic form of E(Q_3_)_indoline_ among three different chemical states is a catalytically important state (Figure 3b); however, this could not sufficiently explain the abnormal ^17^O chemical shift values in this study. Subsequent quantum chemical calculations, along with the experimental chemical shift data of several intermediates, concluded that the percentage of the acid form was noticeably lower than that obtained using the previously proposed method, whereas the experimental observation was more consistent with the predominance of the phenolic tautomer.

**Figure 4:**
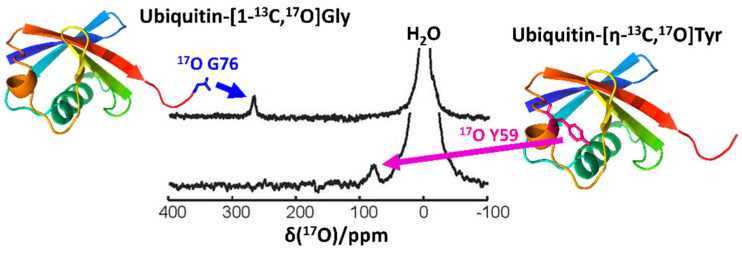
The solution-state ^17^O NMR spectra of ubiquitin-[1-^13^C,^17^O]Gly (top) and ubiquitin-[η-^13^C,^17^O]Tyr (bottom) measured at 16.4 T. The residues whose signals were observed in the corresponding spectra were noted in the structural model of ubiquitin (PDB 1UBQ). The figure was reproduced with permission from [20].

Another recent and important advancement in the field of biological ^17^O NMR spectroscopy is the development of an efficient ^17^O isotope labeling protocol for biomolecules, which remains a challenge to overcome. Notably, Lin et al. [20] recently reported an effective procedure incorporating ^17^O in an amino-acid-specific way to yeast ubiquitin, which was produced recombinantly in *E. coli* (Figure 4). By cultivating auxotrophic strains of *E. coli* in a minimal medium supplemented with ^17^O-incorporated amino acids [9,55], they successfully demonstrated ^17^O labeling at the Gly, Tyr, and Phe residues of yeast ubiquitin. Notably, they were able to observe sharp ^17^O signals from the carboxylate group of the C-terminal G76 (Figure 4, top). This implied that the C-terminal carboxylate group was in fast local motion, thus placing it in the ω_o_τ_c_ < < 1 regime [19]. On the other hand, the signals from backbone oxygens of glycine residues were only observable in the 20% glycerol, where the backbone oxygen atoms could cross over the regime of ω_o_τ_c_ > > 1. Furthermore, this approach was effective for observing the ^17^O signal from a side chain of Tyr (Figure 4), thereby opening a new gateway for measuring the p*K*_a_ of the hydroxyl group of Tyr with a novel probe and for studying its structural and functional characteristics in both aqueous and solid states.

## 3. Conclusions and Future Directions

Despite receiving less attention than other NMR spectroscopic applications, ^17^O NMR spectroscopy has evident advantages and great potential to cover a wide range of applications, which may contribute to resolving various challenges in biomolecular studies. For example, hydrogen bonding is one of the most important non-covalent interatomic interactions modulating biomolecular structures and functions, yet its direct characterization is still a challenging task, particularly because of the shortage of general and efficient experimental techniques [56]. Along with X-ray and neutron crystallographic approaches [57,58], NMR spectroscopy has been a major tool for investigating the atomistic details of various hydrogen bonds; however, it has often been inferred from indirect observations, such as chemical shift perturbations [59]. As many biomolecular hydrogen bonds are mediated by oxygen atoms, ^17^O NMR spectroscopic approaches may provide direct and sensitive information for the accurate characterization of hydrogen bonds. In particular, as discussed above, direct measurement of the p*K*_a_ of oxygen-containing groups in biomolecules can be a highly promising approach not only for hydrogen bond characterization but also for speculating its physiological features.

On the other hand, it should be noted that most biomolecules are surrounded by an excess amount of water molecules, and many physiological processes are dependent on their active participation. In particular, proteins often harbor several internal water molecules within their hydrophobic core, for versatile purposes such as stability modulation, functional regulation, catalysis, transport, and interaction with other biomolecules [60,61,62,63]. The detailed appreciation of this order of water is sometimes critical for modulating biomolecular interactions or designing novel drug molecules targeting water-filled sites [64,65,66]. One representative example includes the X-ray crystallographic study of the human A_2A_ adenosine receptor bound to its endogenous agonist adenosine, where several ordered water molecules were found with adenosine around the ligand-binding cavity [67]. In addition, hemoglobin, the hetero-tetrameric oxygen transporter, is a well-known example of water molecules in its subunit interfaces [62,68]. Ligand binding to this protein strongly affects the ordered water cluster at the subunit interface, which may contribute to allosteric conformational changes in hemoglobin [68,69]. ^17^O NMR spectroscopy is a promising approach to reveal the interaction network and dynamic features of these water molecules. Also, understanding the physiological events associated with these structured water molecules may contribute to developing novel modulation strategies for therapeutic purposes. 

One notable advancement in the field of ^17^O NMR spectroscopy is, as discussed above, the development of novel ^17^O incorporation protocols for proteins [20]. Extension of the current amino-acid-specific labeling protocol to additional versatile procedures enabling more diverse labeling schemes will be an important step to widen the application range of ^17^O NMR techniques. In particular, heteronuclear correlation spectroscopy of ^17^O with other nuclei, such as ^1^H, ^2^H, ^13^C, and ^15^N, may become an invaluable tool for appreciating biomolecular structures and functions that could not be elucidated before. Likewise, the development of novel ^17^O labeling procedures may also have a profound impact on other spectroscopic studies, such as electron paramagnetic resonance and infrared spectroscopy, concomitantly advancing our fundamental understanding of biomolecules. We believe that, together with ongoing theoretical and technical progress, ^17^O NMR spectroscopy may soon become a unique and indispensable tool for characterizing biomolecules.
